# Research on the Preparation of High Molecular Weight Natural Rubber and Its Structure–Property Relationship

**DOI:** 10.3390/polym18111382

**Published:** 2026-06-02

**Authors:** Xinlei Zhang, Meijuan Jiang, Hao Zhang, Yulong Jing, Xin Zhao, Quan Sun, Qinghua Liu, Youxuan Wang

**Affiliations:** Shandong Institute of Non-Metallic Materials, Jinan 250031, China

**Keywords:** natural rubber, high molecular weight, microstructure, structure–property relationship

## Abstract

This work used the same batch of collected natural latex as raw materials in order to ensure the same non-rubber components were contained in natural rubber. Through systematic testing of the rubber material, including microstructure, intrinsic properties and application performance, the preparation process of high molecular weight natural rubber and its structure–property relationship were explored. Additionally, a comparative analysis was conducted with Indonesian No. 1 Ribbed Smoked Sheet (RSS) rubber. The results indicated that regulating the maturation time made an effective contribution to the increasing of molecular weight in natural rubber, with non-rubber components kept constant. The typical mechanical properties such as tensile modulus and tensile strength at room temperature, as well as application performance including hardness and torsion fatigue life of natural rubber were significantly optimized, accompanied by the higher molecular weight. This work successfully prepared high molecular weight natural rubber with superior comprehensive performance and revealed the underlying mechanism between molecular weight and the application properties of natural rubber. It provides a theoretical basis and technical support for the high-performance preparation of natural rubber with application under complex working conditions such as dynamic loading and high-temperature environments.

## 1. Introduction

As an excellent natural polymer material, natural rubber plays an irreplaceable role in the national economy and military fields. Its strain-induced crystallization characteristic makes it a self-reinforcing rubber [1,2], and its outstanding mechanical properties such as high impact resistance, tear resistance, puncture resistance, and fatigue resistance are incomparable to many synthetic rubbers [3]. It directly affects the combat effectiveness and operational reliability of equipment, serving as a crucial strategic resource [4,5]. Natural rubber mainly consists of rubber hydrocarbon and non-rubber components [6]. Its performance is primarily determined by the molecular weight and the distribution of rubber hydrocarbon, as well as the type and content of non-rubber components such as proteins and lipids. As a typical polymer material, the comprehensive performance of natural rubber is closely related to its molecular weight and distribution, which are key factors governing its processability and the performance of final products [7]. High molecular weight natural rubber exhibits excellent physical and mechanical properties, demonstrating superior tear resistance, thermal stability, and fatigue durability under extreme conditions such as high temperature, high dynamic load, and strong impact. Thus, it is one of the critical materials for achieving breakthroughs in equipment reliability and service life in the military field [8,9,10]. However, natural rubber has a complex multi-component system, and its final performance is affected by both molecular weight and non-rubber components such as proteins and lipids [4,10,11]. Most previous studies failed to eliminate the interference of non-rubber components. Therefore, systematic research that independently reveals the effect of molecular weight on the structure and properties of natural rubber is still insufficient, which restricts the accurate regulation and high-performance preparation of natural rubber materials. The molecular weight of natural rubber, raw rubber and the content of fractions with different molecular weights can be regulated through adjustments of processing technologies and chemical methods, including coagulation method and time control, moisture content control, sheet hanging and air-drying time control, and drying temperature and time control [12]. This enables the preparation of high molecular weight and gelled natural rubber to meet the specific requirements of high-performance rubber products.

This study has shown that the high load-bearing capacity and the high wear resistance and tear resistance of rubber materials can be achieved by controlling the content of high and low molecular weight segments in raw natural rubber [13,14]. Moreover, these materials maintain good processability even with ultra-high molecular weight fractions, making them suitable for the production of high-performance rubber products to meet the growing demands of technological development. Currently, Indonesian No. 1 RSS rubber is widely used in high-performance fields [15], while there is a lack of customized natural rubber with tailored molecular weight and distribution based on high-performance requirements. At the raw rubber level, it is necessary to design the micro-mesoscopic component structure centered on molecular weight and distribution, address the insufficient refinement in traditional raw rubber development, improve the stability of the raw rubber component structure, and clarify the regulatory mechanism [16]. In this study, using the same batch of natural latex as raw material and ensuring consistent non-rubber components, natural rubber samples with different molecular weights were prepared by adjusting the maturation time. The content of high molecular weight fractions in raw natural rubber was regulated to achieve structuralization and high molecular weight modification. Through systematic testing of the microstructure and intrinsic properties, physical properties, environmental performance, and application performance of raw rubber, the structure–property relationship of high molecular weight natural rubber was thoroughly explored, and the influence of high molecular weight on the properties of natural rubber was revealed. This provides a theoretical basis for the preparation of high-performance natural rubber, facilitates its application breakthroughs in harsh working conditions such as military fields, and realizes the high performance and customization of high-performance natural rubber materials.

The scientific novelty of this work is that by controlling the maturation time and maintaining consistent non-rubber components, the independent effect of molecular weight on rubber properties can be exclusively investigated, and the precise structure–property relationship is clearly revealed. In addition, it is confirmed that the prepared high molecular weight natural rubber exhibits better application performance than commercial Indonesian No. 1 RSS rubber. This study provides a feasible technical route for the controllable preparation and customized application of high-performance natural rubber.

## 2. Experimental Section

### 2.1. Reagents and Instruments

Grade 1 RSS rubber was supplied by Thai Hua Rubber Public Company Limited (Bangkok, Thailand). Samples were labeled D0, D10, D20, D30, and D40 according to different maturation times. Other reagents, rubber fillers, and additives were commercially available industrial products. The instruments and equipment used in this study are shown in Table 1.

### 2.2. Experimental Procedures

Fresh latex was screened to remove impurities, mixed uniformly, and the dry rubber content was measured. After the latex coagulated, residual impurities were removed by washing, and the rubber was cut into uniform blocks. The rubber blocks were pressed using a creping machine to remove moisture and expel air bubbles, forming thin rubber sheets. The thin rubber sheets were cured in a cool and ventilated place for 0 days, 10 days, 20 days, 30 days, and 40 days, respectively. After maturation, the sheets were hung in a smoke house and smoked with wood smoke (temperature controlled between 40 and 60 °C) for 3–5 days. Finally, the sheets were trimmed and packaged into finished products.

Two formulations were used for evaluating application performance: a test formulation and a laboratory evaluation formulation for rubber bushings. The test formulation (parts by mass) was: NR 100, zinc oxide 5, stearic acid 2, N330 carbon black 50, accelerator TBBS 0.7, and sulfur powder 2.25. The laboratory evaluation formulation (parts by mass) was: natural rubber 100, zinc oxide 3–5, stearic acid 2–3, carbon black reinforcing filler 50, antioxidant 4010NA 2–3, sulfur 2–3, and accelerator NS 0.5–1.5.

The compound rubber was prepared using a two-stage mixing process. In the first stage, the initial temperature of the internal mixer was set to 50 °C with a rotational speed of 60 min^−1^. Natural raw rubber was added and mixed for 60 s, followed by the addition of small ingredients such as zinc oxide and stearic acid for 60 s of mixing. Carbon black reinforcing filler was then added and mixed for 120 s before discharge. In the second stage, sulfur was added using an open two-roll mixer. After uniformly mixing sulfur with accelerators, the compound was folded into five triangular packets, then sheeted and stored.

The compounded rubber was vulcanized using a plate vulcanizing press at 145 °C for 20 min.

### 2.3. Testing and Characterization

(1) Microstructure of Raw Natural Rubber

Determination of molecular weight and distribution: Tests were performed in accordance with ISO 16564-2004. A 0.1 g sample was taken from different positions of the raw rubber, dissolved in 30 mL of tetrahydrofuran (chromatographic grade), shaken for 1 min, and stored in the dark at 37 °C for 24 h. The supernatant was filtered through a 0.45 μm filter membrane, and tests were conducted using a gel permeation chromatograph at a test temperature of 40 °C and a flow rate of 1 mL/min.

Nitrogen content determination: Samples were digested in accordance with GB/T8088-2008, and nitrogen content was tested using the inductively coupled plasma (ICP) method.

Determination of elements (Ca, Cu, Fe, K, Mg, Mn, Na, Zn, P, Si): After drying and grinding the sample, a precise amount was weighed and placed in a polytetrafluoroethylene digestion tank. A mixed acid system of nitric acid and hydrogen peroxide was added for microwave digestion, and the solution was volumetrically adjusted after cooling. The resulting solution was tested using an inductively coupled plasma emission spectrometer (ICP-OES) in accordance with GB/T 41945-2022.

(2) Physical and Mechanical Properties of Vulcanized Rubber

Tensile properties were tested in accordance with GB/T528-2009, with high-temperature tensile tests conducted at 100 °C. Tear properties were tested in accordance with GB/T529-2008. Shore A hardness was tested in accordance with GB/T531.1-2008. Compression fatigue heat build-up properties were tested in accordance with GB/T1687.3-2016. Hot air aging properties were evaluated by aging the samples at 100 °C for 24 h in accordance with GB/T3512-2014, followed by tensile property tests in accordance with GB/T528-2009. The rubber ring torsion fatigue was tested with the load set to 2800 kg, rotational speed at 256 rounds/minute, and breaking deformation to 2.8 mm. The number of torsions at break was recorded as the fatigue life.

## 3. Results and Discussion

### 3.1. Microstructure of Raw Rubber

The molecular weight and distribution of natural rubber are important microstructural parameters determining its processability and mechanical properties. Different maturation times can induce the structuring of natural rubber hydrocarbon chains, thereby altering their molecular weight characteristics and further influencing the viscoelastic and macro-mechanical behaviors of the material. Additionally, the properties of natural rubber are closely related to its non-rubber components. Protein degradation, phospholipid migration, and the redistribution of metal ions during maturation affect the interactions between rubber molecular chains and the formation of gel networks, leading to changes in the macro-performance of the material [17,18]. In order to comprehensively reveal the influence of maturation time on the microstructure of natural rubber, multiple structural analyses were conducted, including the relative molecular weight and distribution, nitrogen content, and phosphorus content of samples under different maturation times.

**Figure 1:**
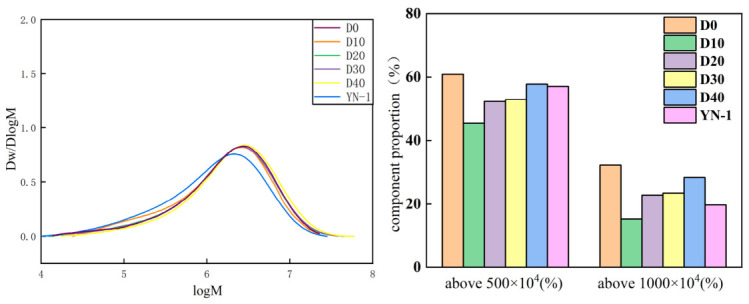
Molecular weight distribution; high molecular weight proportion.

The data obtained from gel permeation chromatography were processed manually to minimize errors caused by integration range and software version. A standard curve was used to convert retention time to logM with the range set to 4–7.5, and the baseline was defined as the line connecting two points where the chromatogram flattened at both ends. The processing results are shown in Figure 1, and the molecular weight data are presented in Table 2.

As shown in Table 2, the number-average molecular weight (Mn) of natural rubber generally increased with prolonged maturation time. The initial sample (D0) had a relatively low Mn, which fluctuated slightly after 10 days of maturation (D10). However, when cured for 20 days or more (D20, D30, D40), Mn was increased significantly and stabilized. This indicates that the molecular chains of raw natural rubber undergo structuring during maturation, forming a macromolecular branched network, which effectively reduces the proportion of low molecular weight fractions and promotes the structuring reaction of molecular segments. Compared with Indonesian No. 1 RSS rubber (YN-1), all self-prepared samples exhibited higher Mn than YN-1, with a more pronounced advantage observed in samples cured for 20 days or more. This confirms that high molecular weight natural rubber can be effectively prepared by regulating. The weight-average molecular weight (Mw), a key indicator reflecting the proportion of high molecular weight fractions, showed a positive correlation with maturation time [19]. From D0 to D40, Mw was increased gradually, reaching a peak in the D40 sample. This suggests that extending maturation time continuously promotes the formation and accumulation of high molecular weight fractions, consistent with the variation trend of Mn. In contrast, the Mw of the reference sample YN-1 was significantly lower than that of all self-prepared samples, indicating the successful preparation of natural rubber with more prominent high molecular weight characteristics through maturation time regulation. Overall, the PDI values of samples D0–D40 ranged from 4.66 to 6.78 and showed a general downward trend, revealing a regular variation in molecular weight distribution. A lower PDI value corresponds to a narrower molecular weight distribution and better uniformity of molecular chain length. In comparison, YN-1 exhibited a PDI value of 10.35 with the broadest molecular weight distribution, indicating a significant difference in molecular chain length.

As shown in Figure 1, the D0 sample had the highest proportion of natural rubber hydrocarbon chains with a molecular weight exceeding 5 million. From D10 to D40, the proportion of molecular chain segments with molecular weights exceeding 5 million and 10 million increased sequentially. Compared with Indonesian Factory 7 RSS rubber, the weight proportion of molecular chain segments with molecular weights exceeding 10 million in D20, D30, and D40 samples all exceeded that of the Indonesian counterpart.

High molecular weight fractions with molecular weights exceeding 500 × 10^4^ are the core structural basis for the mechanical and application properties of natural rubber. These fractions provide key support for the tensile modulus at a given elongation and tensile strength of the material by enhancing the entanglement density of molecular chains. The superior mechanical strength was demonstrated thereby with the denser entanglement of molecular chains, the greater the sliding resistance of chain segments under external force, and the stronger the material’s ability to resist plastic deformation [20]. Meanwhile, the dense entanglement network of high molecular weight segments can also delay the degradation and disentanglement of molecular chains under thermal action, effectively improving the thermal stability of the material and enabling it to maintain structural integrity under high-temperature conditions [21]. Ultra-high molecular weight fractions with molecular weights exceeding 1000 × 10^4^ play a crucial role in enhancing high load-bearing performance. When materials are subjected to dynamic cyclic loads, ultra-high molecular weight chain segments can suppress the initiation and propagation of fatigue cracks through their own “elastic buffering” and “energy dissipation” mechanisms, significantly enhancing the service life of the materials under extreme stress conditions [22]. Statistics on the content of fractions with molecular weights exceeding 5 million and 10 million are presented in Figure 1. As shown in Figure 1, the proportion of both fractions was highest in D0, decreased in D10, and then gradually increased with prolonged maturation time. After 20 days of maturation, the proportions of both fractions in the samples exceeded those in YN-1.

The nitrogen content of raw rubber under different maturation times was basically the same, ranging approximately from 0.5 to 0.6, without showing a regular increasing trend. This indicates that it can effectively control the protein content in raw rubber by mixing the same batch of latex before sheet formation. The ash content and volatile matter did not show regular changes and fluctuated slightly, not exceeding 0.4 and 0.5 respectively, which met the general inspection standards. The D40 sample, with the longest maturation time, had the highest Mooney viscosity, while the other samples ranged from 95 to 100. This may be related to changes in molecular weight; as maturation time increases, a certain degree of molecular chain entanglement or micro-crosslinking occurs, leading to an increase in Mooney viscosity.

As shown in Table 3, the contents of K and Na elements generally decreased first and then increased, while other elements fluctuated within a small range without obvious regularity. During the maturation process of natural rubber, the content of various elements may fluctuate due to impurities from natural sources and external contaminants.

By mixing the same batch of latex after collection to control non-rubber components and then preparing RSS rubber with different maturation times, the resulting samples exhibited approximately the same nitrogen content, ash content, volatile matter content, and element content. In contrast, the molecular weight and the proportion of high molecular weight fractions increased with prolonged maturation time. Compared with the commercially available YN-1 sample, this indicates that high molecular weight natural rubber was successfully prepared under the premise of controlling other micro-components to be consistent. Natural rubber with different molecular weights, including high molecular weight natural rubber, can be prepared by adjusting the maturation process.

### 3.2. Structure–Property Relationship of Natural Rubber

#### 3.2.1. Vulcanization Characteristics with Testing Formulation

The vulcanization characteristics of rubber mainly include the initial vulcanization time, optimal vulcanization time, and vulcanization process, etc. Among them, T_10_ represents the time for rubber vulcanization to reach 10%, which is the delayed action time before the start of hot vulcanization, equivalent to the induction period of the vulcanization reaction, reflecting the anti-vulcanization scorching characteristics of the mixed rubber [23]. T_90_ represents the time for the rubber compound to vulcanize to reach 90%, which is the optimal vulcanization time of the process. The minimum torque can reflect the processing characteristics and quality consistency of the rubber compound during the vulcanization process. The maximum torque can reflect the modulus level of rubber materials.

The vulcanization characteristics of raw natural rubber with different molecular weights were investigated using the test formulation under the same mixing process. As shown in Table 4, the regulation of vulcanization characteristics by natural rubber prepared with different maturation times exhibited a mild and directional trend overall. In terms of the vulcanization reaction process, natural rubber raw materials with different molecular weights showed similar vulcanization characteristics. Different maturation times did not affect the vulcanization processability of raw natural rubber, indicating good processing and application properties. The maximum torque (MH) of vulcanized high molecular weight natural rubber (D20, D30, D40) prepared with the test formulation increased significantly, reflecting that the increase in the molecular weight of natural rubber led to a higher crosslink density of the vulcanized network, laying a structural foundation for enhancing the load-bearing capacity of the vulcanized rubber.

#### 3.2.2. Mechanical Properties with Testing Formulation

The hardness of vulcanized rubber and the 100% and 300% tensile modulus at a given elongation can directly reveal the magnitude of the modulus of natural rubber materials under different force conditions, reflecting the load-bearing capacity of natural rubber during use. The tensile strength of the sample can reflect the ultimate ability of natural rubber materials to resist tensile failure. High-temperature tensile strength represents the ability of a sample to resist damage in a high-temperature usage environment and can reflect the performance of rubber materials during product use to a certain extent. Elongation at break can reflect the maximum deformation of rubber materials, resilience can reflect the elastic deformation capacity of rubber materials, and tear strength can evaluate the ability of rubber materials to resist defects and crack propagation. 300% tensile modulus at a given elongation and hardness are both important indicators for characterizing the stiffness of vulcanized rubber. Tensile modulus at a given elongation corresponds to tensile deformation, while hardness corresponds to compressive deformation. A higher tensile modulus at a given elongation and hardness indicates a higher elastic modulus and better wear resistance of the compound.

**Figure 2:**
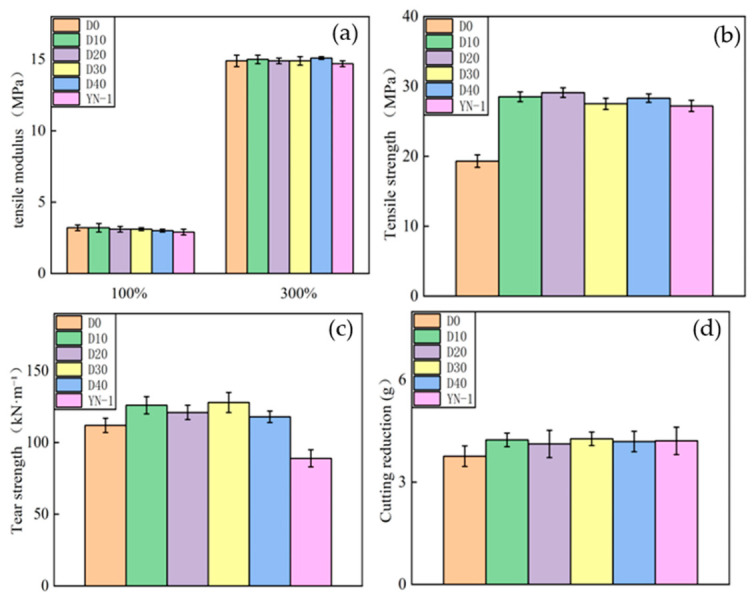
(**a**) Tensile modulus, (**b**) tensile strength, (**c**) tear strength, (**d**) cutting reduction.

The stress under high deformation is highly correlated with the proportion of high molecular weight fractions. A higher proportion of high molecular weight fractions results in a denser structured network of molecular chains, increasing the resistance to segmental sliding under external forces. This corresponds to the significantly higher stress under high deformation in Figure 2a for the self-prepared samples compared to YN-1 (which has a low proportion of high molecular weight fractions and a low degree of molecular chain structuring). Tensile strength is the material’s ability to resist damage when it breaks, which depends on the length of high molecular weight chains and the entanglement strength: higher molecular weight and a greater proportion of high molecular weight fractions result in stronger intermolecular forces and chain entanglement resistance that need to be overcome during the breaking process. As shown in Figure 2b, the tensile strength of D10–D40 samples was generally superior to that of YN-1, attributed to the increased molecular weight and proportion of high molecular weight fractions through maturation regulation. In contrast, D0 had lower tensile strength, corresponding to its low proportion of high molecular weight fractions in the early stage of maturation.

The essence of tear strength is the material’s resistance to crack propagation, where ultra-high molecular weight fractions above 1000 × 10^4^ play a key role. Long-chain molecules can hinder further crack propagation through “cross-crack entanglement,” and longer chains with higher proportions require more energy for crack propagation. As shown in Figure 2c, the tear strength of D0–D40 samples is significantly higher than that of YN-1, consistent with the recovery of the proportion of ultra-high molecular weight fractions in these samples. In contrast, YN-1 has low tear strength due to its low proportion of ultra-high molecular weight fractions, allowing cracks to propagate rapidly. The cut loss generally reflects the wear and cut resistance of the material, which is directly related to the toughness of the dense network formed by high molecular weight fractions. The cut loss of the six samples was approximately the same, indicating that the five self-prepared samples with different maturation times exhibited excellent cut resistance comparable to YN-1, meeting the requirements of harsh working conditions.

#### 3.2.3. Vulcanization Characteristics of the Application Formulation

Natural rubber needs to be crosslinked through vulcanization to meet engineering applications, and the microstructural characteristics of different natural rubbers affect the subsequent vulcanization process. Therefore, the vulcanization characteristics of raw natural rubber samples with different molecular weights were studied using a commonly used industrial formulation in this study. As shown in Table 5 from the early to middle stages of maturation (i.e., D0–D30), T10 and T90 exhibited little fluctuation, and the vulcanization characteristics of all samples met the requirements of processing safety. Raw high molecular weight rubber has long molecular chains and dense entanglement, resulting in higher network strength after crosslinking and a significant increase in MH. As shown in Table 5, the MH of D0–D40 samples was higher than that of YN-1, with the D40 sample (exhibiting the most prominent high molecular weight characteristics) achieving the highest MH. Combined with the MH-ML value, it can be indicated that the crosslink density was higher in the late stage of maturation, consistent with the molecular weight test results. The loss factor (Tan θ) of each sample was approximately the same, indicating good molecular chain regularity and a uniform crosslink network.

#### 3.2.4. Mechanical Properties of the Production Formulation

The tensile properties of different compounds were measured at room temperature using a tensile testing machine, and the results are listed in Table 6. The tensile stress at a given elongation increased with increasing molecular weight and the proportion of high molecular weight fractions. The tensile behavior of D40 was consistent with this trend, supporting the positive effect of high molecular weight fractions on tensile deformation. Tensile strength and elongation at break also showed an increasing trend with the elevation of molecular weight and the content of high molecular weight fractions, which was related to the longer molecular chains and higher entanglement density. With the prolongation of maturation time, the tensile properties exhibited a stepwise evolution. The samples at the early maturation stage tended to have higher elongation at break, while those at the late stage were characterized by enhanced stiffness and tear resistance. Overall, the tensile mechanical properties evolved in a time-dependent manner during maturation. The changes were consistent with the evolution of the microstructure of raw rubber, and a general upward trend was observed with increasing molecular weight.

High-temperature tests were conducted at 100 °C, as shown in Table 7. Compared with the room-temperature mechanical properties, the tensile modulus at high temperature slightly increased with prolonged maturation time and then stabilized, with small fluctuations, and was superior to that of YN-1 overall. The tensile strength and elongation at break gradually decreased, indicating that prolonged maturation increased the molecular weight and molecular chain entanglement of natural rubber [24]. High temperature intensified the thermal motion of molecular chains, weakened the intermolecular forces, and made the segments more prone to slippage under stress, thereby leading to a gradual reduction in toughness [25,26]. However, the decrease was controllable, and the material could still meet the requirements of medium- and low-temperature heat-resistant applications.

Mechanical properties were tested after aging in hot air at 100 °C for 24 h, as shown in Table 8. The tensile modulus after aging did not show an obvious regular pattern, fluctuating only within a small range, and the aging properties of all samples exhibited similarity [27].

#### 3.2.5. Compression Fatigue Heat Build-Up Characteristics

**Figure 3:**
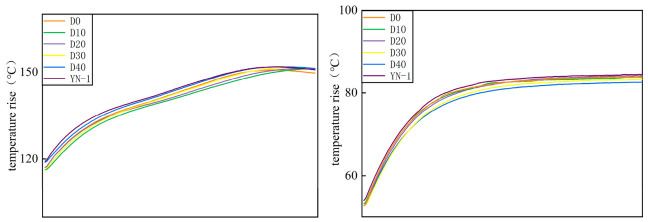
Compression heating temperature rise curve at 55 °C/120 °C.

Compression heat generation characteristics were tested under constant load mode at 55 °C and 120 °C, and the results are shown in Figure 3. As shown in Figure 3, the D40 sample with the higher molecular weight exhibited the lowest temperature rise. The temperature rise rule showed that the lower the molecular weight, the higher the temperature rise. The branched network formed by the structuring of high molecular weight natural rubber molecular chains is tougher. Under load, the segments can better offset the force through rebound. Additionally, the density and uniformity of the branched network result in higher hardness, further enhancing its load-bearing capacity. This further verifies that under the condition of approximately the same other components, natural rubber with high molecular weight and a high proportion of high molecular weight fractions has better load-bearing capacity [28,29,30]. Compared with the heat generation performance of YN-1, the D series samples generally exhibited lower heat generation with flatter curves than that of YN-1.

#### 3.2.6. Product Bench Test Durability

**Figure 4:**
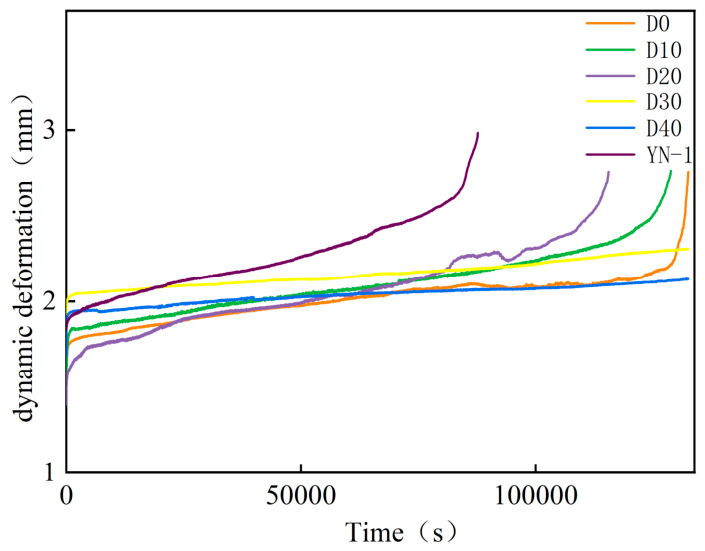
Fatigue temperature rise curve; stiffness variation curve.

The actual working conditions of the rubber compound were simulated by using a rubber ring torsional fatigue testing machine, and the results are shown in Figure 4 and Table 9. As shown in Figure 4, the fatigue life of the D series samples generally showed an increasing trend, while the change in deformation gradually slowed down, the product hardness gradually increased, and the temperature rise decreased. The D40 sample exhibited the highest fatigue life, the highest hardness, and the lowest temperature rise. The higher fatigue life and slower increase in deformation are attributed to its high molecular weight and a large proportion of high molecular weight fractions, resulting in dense molecular chain entanglement and a strong elastic network resistance to deformation [31,32]. Under stress, the segments are not prone to sliding, demonstrating better load-bearing capacity. In contrast, the deformation curve of YN-1 was steeper with more obvious changes, due to its low molecular weight and a small proportion of high molecular weight fractions, resulting in sparse molecular chain entanglement and easy irreversible sliding of segments under stress, leading to weak structural stability. The product hardness reflects the density and uniformity of the molecular chain crosslink network: a denser and more uniform network formed after crosslinking results in better consistency and stability of hardness. Low molecular weight and sparse molecular chains lead to increased friction between molecular chains and higher internal heat generation under high load, which is particularly evident in fatigue tests: YN-1, with low molecular weight and a small proportion of high molecular weight fractions, exhibited the highest temperature rise. Overall, the D series natural rubber was superior to YN-1 in terms of deformation control, hardness stability, and temperature rise optimization. The core reason is the enhanced molecular chain entanglement and more stable elastic network brought about by the increased molecular weight and optimized proportion of high molecular weight fractions, further verifying the successful preparation of high molecular weight natural rubber.

## 4. Conclusions

In this study, natural rubber with controllable molecular weight was prepared by adjusting maturation time using the same batch of natural latex while keeping non-rubber components consistent. With the extension of maturation time, the number-average molecular weight increased from 339,097 g/mol to 674,815 g/mol. The weight-average molecular weight of D40 reached a maximum of 3,469,867 g/mol, accompanied by a narrowed molecular weight distribution.

Prolonged maturation time enhances molecular chain entanglement and network compactness, which effectively increases the molecular weight of natural rubber. The increase in molecular weight remarkably improves the overall properties and modulus of vulcanizates. At room temperature, the 300% tensile modulus and tensile strength of vulcanizates are obviously optimized. The tensile service performance at 100 °C remains stable and is superior to commercial YN-1 rubber.

After thermal-oxidative aging, the mechanical properties of the material decline in a controllable range. In particular, the torsion fatigue life of D40 reaches 107.9 × 10^4^ cycles, much higher than the 37.5 × 10^4^ cycles of YN-1 rubber, presenting lower compression heat build-up and better dynamic stability. This work reveals the structure–property relationship of high molecular weight natural rubber under maturation regulation, providing a solid theoretical basis and technical support for the preparation and customized application of high-performance natural rubber.

## Figures and Tables

**Table 1:** Instruments and equipment.

Experimental Equipment	Models	Manufacturer
Open two-roll mill	X(S)KG160	Shanghai Rubber Machinery Factory (Shanghai, China)
Small intelligent internal mixer	BR1600	Farrell Company (Ansonia, CT, USA)
Plate vulcanizing press	TYG100	Dong Yu Hydraulic Machinery Co., Ltd. (Nantou, China)
GPC	PL-GPC 220	Agilent Technologies (Santa Clara, CA, USA)
Centrifuge	CR21N	Hitachi Scientific Instruments (Tokyo, Japan)
Automatic Kjeldahl nitrogen analyzer	KYG3220DG160	Shanghai Yihong Instrument Co., Ltd. (Shanghai, China)
ICP-OES	ICP6300	Thermo Fisher Scientific (Waltham, MA, USA)
Mooney viscometer	M200E	Beijing Youshen Electronic Instrument Factory (Beijing, China)
Rotorless curemeter	C2000E	Beijing Youshen Electronic Instrument Factory (Beijing, China)
MTS universal testing machine	/	MTS Industrial Systems (China) Co., Ltd. (Shanghai, China)
Aging test chamber	LRG01A	Chongqing Huida Test Instrument Co., Ltd. (Chongqing, China)
Rubber ring torsion fatigue tester	JNPG50	Jinan Jingcheng Testing Technology Co., Ltd. (Jinan, China)

**Table 2:** Molecular weight of natural rubber.

Sample	Mn (g/mol)	Mw (g/mol)	PDI	Nitrogen Content	Ash Content	Matte Volatile	ML 3 + 4 100 °C
D0	339,097	2,298,803	6.78	0.54	0.28	0.16	99.11
D10	385,087	2,569,461	6.67	0.52	0.24	0.20	99.16
D20	664,762	3,099,432	4.66	0.50	0.25	0.21	98.27
D30	671,976	3,146,235	4.68	0.53	0.31	0.25	95.03
D40	674,815	3,469,867	5.14	0.58	0.36	0.23	111.57
YN-1	224,590	2,323,688	10.35	0.55	0.32	0.22	107.86

**Table 3:** Elemental analysis.

Sample	Ca	Cu	Fe	K	Mg	Mn	Na	Zn	P	Si
D0	24	/	24	615	192	/	7.9	2.3	512	/
D10	36	/	11	456	125	/	5.4	1.2	474	/
D20	29	/	12	579	173	/	6.0	1.0	469	/
D30	37	/	35	771	259	/	8.8	2.0	544	/
D40	39	/	10	874	241	/	12.2	2.3	686	/
YN-1	62	/	22.5	707	160	0.8	7.2	2.2	665	/

**Table 4:** Vulcanization characteristics of test formula compounds.

145°	T_10_	T_90_	M_L_	M_H_	Tan θ
D0	3.91	12.38	2.21	18.53	0.05
D10	3.64	12.12	2.51	18.95	0.05
D20	4.11	12.95	2.57	19.50	0.05
D30	3.72	12.51	2.27	19.43	0.04
D40	3.88	12.98	2.55	19.45	0.04
YN-1	3.30	12:18	2.09	18.40	0.04

**Table 5:** Vulcanization characteristics of application formula compounds.

Sample	T10	T90	ML	MH	MH-ML	Tan θ
D0	3.24	8.69	1.87	17.99	16.12	0.04
D10	3.03	8.73	2.23	17.71	15.48	0.03
D20	3.16	8.48	2.17	17.74	15.57	0.03
D30	3.18	8.32	1.88	18.13	16.25	0.03
D40	3.28	8.99	2.24	18.32	16.08	0.03
YN-1	3.19	8.56	1.66	15.22	13.56	0.03

**Table 6:** Room-temperature mechanical properties of vulcanized rubber.

Properties	D0	D10	D20	D30	D40	YN-1
100% tensile modulus [MPa]	3.1 ± 0.2	2.9 ± 0.1	2.9 ± 0.3	3.1 ± 0.2	3.2 ± 0.2	3.2 ± 0.2
200% tensile modulus [MPa]	7.3 ± 0.1	6.8 ± 0.2	6.8 ± 0.2	7.4 ± 0.1	7.5 ± 0.1	6.7 ± 0.2
300% tensile modulus [MPa]	12.9 ± 0.1	11.9 ± 0.2	12.1 ± 0.2	12.9 ± 0.1	12.9 ± 0.2	11.2 ± 0.3
Tensile strength [MPa]	26.3 ± 0.7	24.8 ± 0.9	26.5 ± 0.9	26.9 ± 1.1	27.3 ± 0.8	26.2 ± 0.9
Elongation at break [%]	584 ± 28	514 ± 33	534 ± 29	538 ± 32	540 ± 25	510 ± 36
Permanent set [%]	32 ± 2	30 ± 4	28 ± 4	16 ± 3	34 ± 5	32 ± 5
Shore A hardness	66	66	66	68	66	67
Resilience [%]	46 ± 3	46 ± 4	48 ± 4	46 ± 6	47 ± 3	42 ± 5
Tear strength [kN·m^−1^]	49 ± 5	46 ± 5	50 ± 6	56 ± 8	52 ± 5	51 ± 7

**Table 7:** High-temperature mechanical properties of vulcanized rubber.

Properties	D0	D10	D20	D30	D40	YN-1
100% tensile modulus [MPa]	2.4 ± 0.3	2.4 ± 0.2	2.5 ± 0.3	2.5 ± 0.3	2.5 ± 0.2	2.2 ± 0.2
200% tensile modulus [MPa]	4.3 ± 0.2	4.4 ± 0.2	4.6 ± 0.4	4.6 ± 0.2	4.6 ± 0.2	4.1 ± 0.1
300% tensile modulus [MPa]	6.3 ± 0.1	6.5 ± 0.1	6.5 ± 0.2	6.4 ± 0.2	6.7 ± 0.4	5.7 ± 0.3
Tensile strength [MPa]	16.4 ± 0.4	15.2 ± 0.6	14.9 ± 0.7	14.7 ± 0.4	15.7 ± 0.6	14.4 ± 0.7
Elongation at break [%]	770 ± 25	707 ± 32	678 ± 28	673 ± 28	578 ± 25	753 ± 31
Tear strength [kN·m^−1^]	38 ± 3	38 ± 5	36 ± 5	38 ± 6	33 ± 3	38 ± 8

**Table 8:** Aging mechanical properties.

Properties	D0	D10	D20	D30	D40	YN-1
100% tensile modulus [MPa]	4.3 ± 0.2	4.5 ± 0.3	4.3 ± 0.3	4.5 ± 0.4	4.3 ± 0.3	4.1 ± 0.3
200% tensile modulus [MPa]	9.4 ± 0.4	9.7 ± 0.3	10.4 ± 0.5	/	10.3 ± 0.4	10.6 ± 0.5
300% tensile modulus [MPa]	/	/	/	/	/	/
Tensile strength [MPa]	10.9 ± 0.6	11.8 ± 0.5	13.5 ± 0.5	9.6 ± 0.4	17.1 ± 0.6	16.4 ± 0.6
Elongation at break [%]	211 ± 19	218 ± 14	242 ± 18	186 ± 21	293 ± 19	277 ± 24
Permanent set [%]	6 ± 2	6 ± 3	8 ± 3	4 ± 1	12 ± 3	18 ± 3
Shore A hardness	70	72	71	72	72	69
Resilience [%]	48 ± 3	48 ± 5	48 ± 5	48 ± 3	49 ± 5	42 ± 3
Tear strength [kN·m^−1^]	40 ± 4	40 ± 3	39 ± 5	37 ± 4	37 ± 4	46 ± 3

**Table 9:** Fatigue test.

Properties	D0	D10	D20	D30	D40	YN-1
hardness	70	71	71	72	72	71
fatigue cycles (×10^4^)	77.9	49.2	55.2	93.4	107.9	37.5
dynamic deformation	1.60	1.64	1.50	1.50	1.49	1.72

## Data Availability

The original contributions presented in this study are included in the article. Further inquiries can be directed to the corresponding author.

## Abbreviations

Abbreviation	Full Name
NR	Natural Rubber
HMWNR	High Molecular Weight Natural Rubber
Mn	Number-Average Molecular Weight
Mw	Weight-Average Molecular Weight
PDI	Polydispersity Index
GPC	Gel Permeation Chromatography
RSS	Ribbed Smoked Sheet
ML	Minimum Torque
MH	Maximum Torque
T10	Scorch Time (10% Vulcanization Time)
T90	Optimum Vulcanization Time (90% Vulcanization Time)
Shore A	Shore A Hardness
Tan θ	Loss Factor
